# Multivisceral Resection for Suspected Adrenocortical Carcinoma

**DOI:** 10.3390/jcm14207210

**Published:** 2025-10-13

**Authors:** Agata Dukaczewska, Peer I. Gottschalkson, Wenzel Schoening, Robert Oellinger, Knut Mai, Dominik Soll, Johann Pratschke, Frederike Butz, Martina T. Mogl

**Affiliations:** 1 Department of Surgery, Campus Charité Mitte|Campus Virchow-Klinikum, Charité—Universitätsmedizin Berlin, Corporate Member of Freie Universität Berlin, Humboldt-Universität zu Berlin, and Berlin Institute of Health, 10117 Berlin, Germany; agata.dukaczewska@charite.de (A.D.); peer-ingmar.gottschalkson@charite.de (P.I.G.); robert.oellinger@charite.de (R.O.);; 2 Department of Endocrinology and Metabolism, Charité—Universitätsmedizin Berlin, Corporate Member of Freie Universität Berlin, Humboldt-Universität zu Berlin, and Berlin Institute of Health, 10117 Berlin, Germanydominik.soll@charite.de (D.S.)

**Keywords:** adrenocortical carcinoma, adrenalectomy, multivisceral resection, lymphadenectomy

## Abstract

**Background**: Adrenocortical carcinoma (ACC) is a rare and aggressive malignancy. Complete tumor resection (R0) is critical for prognosis and may require multivisceral resection in locally advanced cases. However, data on outcomes after multivisceral resection for ACC remain limited. This study evaluates the perioperative and oncologic outcomes of patients undergoing multivisceral resection for suspected ACC. **Methods**: We retrospectively analyzed 21 patients who underwent multivisceral resection with curative intent for suspected ACC. Three were later diagnosed with other tumor entities (sarcoma, non-small cell lung carcinoma metastasis and ganglioneuroma). The remaining 18 patients with histologically confirmed ACC were compared with 19 patients who underwent isolated adrenalectomy during the same study period. **Results**: Patients undergoing multivisceral resection were significantly younger (*p* = 0.003), had larger (*p* < 0.001) and more advanced tumors according to ENSAT classification (*p* < 0.001). All but one had open surgery; laparoscopic or hybrid approaches were more common in the isolated adrenalectomy group. Multivisceral resections were associated with longer operative times (*p* = 0.002), all required an ICU admission (*p* < 0.001), and had longer hospital stays (*p* = 0.001). Lymphnode metastases were observed only in the multivisceral group (*p* = 0.002). No significant differences were found in complication rates (*p* = 0.081), resection status (*p* = 0.091), progression-free survival (*p* = 0.095), or overall survival (*p* = 0.71). **Conclusions**: Multivisceral resection is a safe and feasible approach in specialized centers and may achieve comparable oncologic outcomes to isolated adrenalectomy, even in patients with more advanced disease. It should be considered when R0 resection is required and technically achievable.

## 1. Introduction

Adrenocortical carcinoma (ACC) is a rare and aggressive malignancy originating from the adrenal cortex, with an incidence of approximately 1–2 cases per million people per year [1]. Complete surgical resection with negative margins (R0 resection) remains the only potentially curative treatment [1]. However, due to the frequently advanced local extent of disease at the time of diagnosis [2], achieving an R0 resection often requires multivisceral resection [3], involving adjacent organs such as the kidney, liver, pancreas, spleen, or diaphragm. Although technically demanding, this approach offers the best chance for durable oncologic outcomes in selected patients [1].

Suspicion of ACC typically arises in the context of large adrenal tumors (>4 cm) [4], rapid tumour progression, radiologic features of necrosis [5,6], signs of local invasion into surrounding organs or vessels, suspected lymph node metastasis [6] or native computed tomography (CT) density > 20 Hounsfield Units (HU) [3]. A thorough patient and family history, along with a comprehensive hormonal workup to assess for cortisol and androgen secretion, is essential [3,4,7]. Known or suspected familial syndromes associated with elevated risk for ACC, such as Li–Fraumeni [8] or Lynch syndrome [9], as well as rapidly progressive Cushing’s syndrome [1], should raise strong suspicion for adrenal malignancy. It is also critical to exclude pheochromocytoma through biochemical testing of metanephrines and normetanephrines [3,4,7]. Around 50% of ACCs are hormonally inactive [10], and in these cases, differentiating ACC from other adrenal or retroperitoneal pathologies, such as metastases or sarcomas, can be particularly challenging [11]. Adrenal biopsy is not recommended, as it may negatively impact outcomes by compromising the likelihood of achieving a subsequent R0 resection [3]. Consequently, when ACC is suspected, surgical intervention may be the only valid management option.

In this study, we evaluate the indications, outcomes, and surgical considerations associated with multivisceral resection in patients with suspected ACC. Furthermore, we compare clinical and tumour characteristics, as well as perioperative and postoperative outcomes, between patients undergoing multivisceral resection and those receiving isolated adrenalectomy for histologically confirmed ACC.

## 2. Materials and Methods

### 2.1. Study Design

This was a retrospective, single-center study conducted at the Tertiary Center for Endocrine Surgery at the Charité—Universitätsmedizin Berlin, Germany.

### 2.2. Patient Demographic and Clinical Data

Patients who underwent multivisceral resection for suspected adrenocortical carcinoma (ACC) with curative intent, as well as all patients who underwent isolated adrenalectomy for ACC between January 2008 and April 2024 at the Tertiary Centre for Endocrine Surgery, Charité—Universitätsmedizin Berlin, were retrieved from a retrospectively collected database. A total of 24 patients who underwent multivisceral resection for suspected ACC were identified. One patient with bilateral ACC resection and two patients with distant metastases who underwent adrenalectomy for symptom control of hypercortisolism without curative intent were excluded. Thus, 21 patients who underwent multivisceral resection were included in the final analysis. In addition, 19 patients who underwent isolated adrenalectomy for ACC within the same study period were identified. One patient with ACC who underwent cholecystectomy for a non-oncologic reason (cholecystolithiasis) was assigned to the isolated adrenalectomy group.

For all patients, data were collected on patient-specific factors (age, sex, body mass index (BMI) and physical status according to the American Society of Anaesthesiologists (ASA) classification), tumour-specific factors (tumour entity, size and location, and hormone production, presence of vascular and/or adjacent organ infiltration, lymphnode metastases), and intraoperative and postoperative outcomes (surgical technique, duration of surgery, resection status, pathological confirmation of organ infiltration, need for blood transfusion, requirement for postoperative intensive care unit (ICU) monitoring, ICU length of stay, and total postoperative hospital stay). Postoperative complications were recorded and classified according to the Clavien-Dindo system [12], with minor complications defined as grades I to IIIa, and major complications as grade IIIb 3b or higher. For patients who underwent multivisceral resection for suspected ACC, the indications and extent of the multivisceral resection were documented. Additionally, for all ACC patients, data were collected on Ki67 index, ENSAT stage (according to the European Network for the Study of Adrenal Tumours (ENSAT) system) [13], and the S-GRAS score [14], which includes ENSAT stage, Ki67 index, resection status, age, and tumour-related symptoms. This study was conducted in compliance with the principles of the Declaration of Helsinki, following approval from the institutional review board (EA1/394/20, 21 January 2021, and Gliedkörgerschaft der Freien Universität Berlin und der Humboldt-Universität zu Berlin Charitéplatz, EA1/097/21, 23 April 2021).

### 2.3. Statistical Analysis

Normally distributed continuous variables are displayed as means (standard deviation, SD), non-normally distributed continuous variables as medians [range] and categorical variables as frequencies. Normality was assessed using Shapiro–Wilk test. The Mann–Whitney U test was used to compare groups for non-normally distributed metric variables, and the two-sided *t*-test was applied for normally distributed metric variables. The Fischer’s exact test was used for group comparison of categorical variables. Overall survival (OS) and progression-free survival (PFS) were estimated using the Kaplan–Meier method. OS was defined as the time from adrenalectomy to death from any cause, while PFS was defined as interval between adrenalectomy and the first postoperative progression in patients with adrenocortical carcinoma. Patients who had neither died nor shown disease progression, or who were lost to follow-up, were censored at the time of their last recorded visit. Individuals without any follow-up data were excluded from both OS and PFS analyses. Survival curves were compared using the log-rank tests. The significance level was set at *p* < 0.05. All statistical analyses were conducted using R (version 024.12.0 + 467; R Foundation for Statistical Computing, Vienna, Austria).

## 3. Results

We included 21 patients who underwent multivisceral resection for suspected adrenocortical carcinoma (ACC) with curative intent. Patient and tumor characteristics are summarized in Table 1. The cohort had a mean age of 42 years (SD 15 years) and consisted of 14 women (66.7%) and 7 men (33.3%). The mean tumor size was 142 mm (SD 54 mm). Hormonal activity was present in 15 patients (71.4%). Lymph node metastases were detected in 6 patients (28.6%). Vascular infiltration was observed macroscopically in 11 (52.4%) and microscopically in 5 patients (23.8%). The results of the Shapiro–Wilk normality test for this patient group are presented in Table A1.

**Figure 1:**
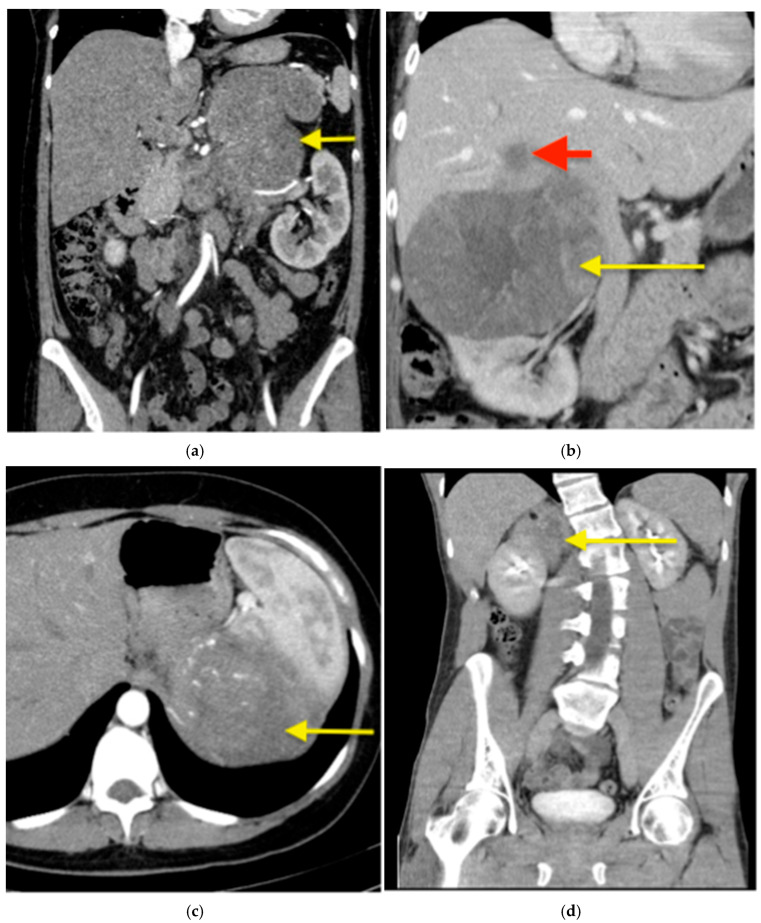
Suspected ACC in computed tomography (CT)—examples. (**a**) A CT with contrast of a 42-year-old female patient with a left sided ACC (arrow) producing cortisol, testosterone and dehydroepiandrosterone, measuring 170 mm in the largest diameter with a suspected infiltration of renal hilum. The adrenal tumour was resected en bloc with the left kidney. (**b**) A CT with contrast of a 49-year-old female patient with a right-sided, cortisol producing ACC (yellow arrow), measuring 150 mm in largest diameter with a liver metastasis in segment VII (red arrow), which was resected en bloc with the adrenal tumour. (**c**) A CT with contrast of a 39-year-old female patient with a hormonally inactive left-sided ACC (arrow), measuring 215 mm in the largest diameter. Preoperatively suspected adhesions to the spleen in the preoperative cross-sectional imaging were intraoperatively loosened. This resulted in a bleeding that required a blood transfusion and ultimately a splenectomy. (**d**) A native CT of a 31-year-old patient with a hormonally inactive tumour (arrow). Infiltration of the kidney by the tumour was suspected both preoperatively on cross-sectional imaging and intraoperatively. A partial nephrectomy was performed en bloc with the adrenal tumour. Histopathological examination confirmed a ganglioneuroma—a benign tumour without kidney infiltration.

The intra- and postoperative outcomes are shown in Table 2. Open surgery was performed in 18 patients (85.7%) and a hybrid approach in 3 patients (14.3%). The median duration of surgery was 236 min [range 102–825 min]. R0 resection was achieved in 16 patients (76.2%). Blood transfusion was required in 6 cases (28.6%). Most patients (20 of 21; 95.2%) required an ICU stay, with a median duration of 1 day [range 1–38]. The median length of postoperative hospital stay was 12 days [range 6–78]. Postoperative complications occurred in 9 patients (42.9%), including 6 minor (28.6%) and 3 major complications (14.3%). The most commonly resected organs were the kidney (*n* = 11; 52.4%) and the liver (*n* = 6; 28.6%). Indications for multivisceral resection included adhesions to adjacent organs (*n* = 4; 19.1%), suspected infiltration of surrounding organs or vessels (*n* = 7; 33.3%), liver metastases (*n* = 4; 19.1%), the need for improved surgical exposure (*n* = 1; 4.8%), and the presence of tumor thrombus (*n* = 1; 4.8%). Of the four patients (19.1%) who underwent multivisceral resection due to suspected organ infiltration, liver involvement was histologically confirmed in two (9.5%) and diaphragmatic infiltration in one (4.8%). No pathological evidence of infiltration was found in the resected kidneys, pancreas, or spleen. In 18 patients (85.7%), the suspected diagnosis of ACC was confirmed histologically. The remaining three patients (14.3%) were diagnosed with other tumor entities: one sarcoma, one adrenal metastasis of non-small cell lung carcinoma, and one ganglioneuroma. Examples of preoperative CT scans of patients with suspected ACC and organ infiltration are presented in Figure 1.

For patients with ACC, patient and tumour characteristics, as well as intraoperative and postoperative outcomes, were compared between the multivisceral resection (*n* = 18, 48.6%) and isolated adrenalectomy (*n* = 19, 51.4%) groups (Table 3 and Table 4, respectively). The results of the Shapiro–Wilk normality test conducted prior to comparative analyses are presented in Table A2. Patients who underwent multivisceral resection were younger and had larger and more advanced tumors. Lymph node metastases were found only in patients who underwent multivisceral resection. Periadrenal lymph node metastases were detected in five (27.8%) patients, and a paracaval lymph node metastasis was observed in one (5.6%) patient.

Tumour hormonal activity did not differ between the groups. The most common hormone excess was combined cortisol and androgen secretion, observed in 5 (27.7%) patients in the multivisceral resection group and 6 (33.3%) patients in the isolated adrenalectomy group. Isolated androgen excess was present in 6 (33.3%) patients in the multivisceral resection group and 1 (5.5%) patient in the isolated adrenalectomy group. Isolated cortisol excess was found in 3 (16.7%) patients in the multivisceral resection group and 3 (16.7%) patients in the isolated adrenalectomy group. Aldosterone secretion was detected in 2 (10.5%) patients in the isolated adrenalectomy group, while no cases were reported in the multivisceral resection group. An overview of hormonal oversecretion by adrenal tumors is presented in Table A3.

All but one patient who underwent multivisceral resection had open surgery (94.4%); one case (5.6%) required conversion from a laparoscopic to an open approach. No procedures in this group were completed laparoscopically. Multivisceral resections had longer operative times compared to isolated adrenalectomies. All patients in this group required postoperative ICU care and had longer hospital stays. There was no statistically significant increase in complication rates in the multivisceral resection group compared to isolated adrenalectomy group.

**Figure 2:**
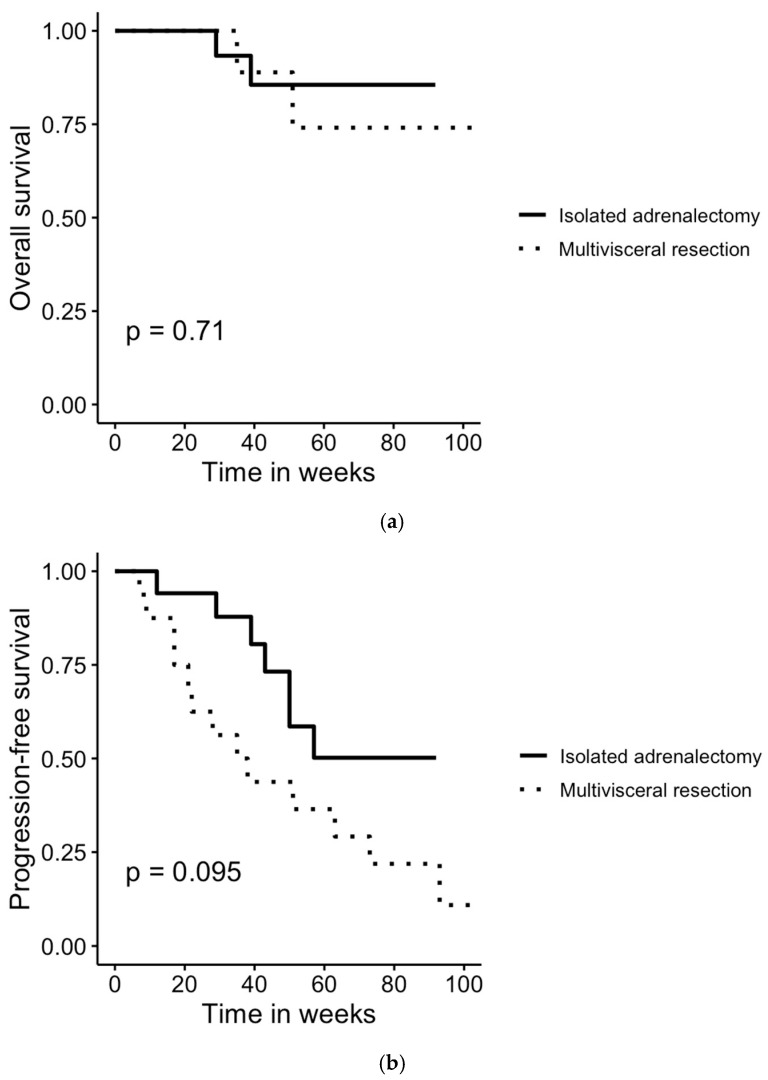
Comparison of (**a**) overall and (**b**) progression-free survival in patients undergoing multivisceral resection and isolated adrenalectomy for histologically confirmed ACC. Survival rates were compared using log-rank test. No statistically significant differences were found between the groups.

Follow-up data were collected in June 2025 and were not available for two ACC patients who underwent multivisceral resection and one patient who underwent isolated adrenalectomy; therefore, these patients were excluded from the survival analysis. Among the ACC patients included in the survival analysis, the median follow-up time was 46.5 months (range: 4–154 months), and the median progression-free survival was 20.5 months (range: 3–154 months). Survival analysis revealed no statistically significant difference in overall survival (OS) between surgical techniques (log-rank *p* = 0.71). The progression-free (PFS) survival was 9.5 months [3–154 months] for patients undergoing multivisceral resections and 31 months [4–92 months] for those undergoing isolated adrenalectomies. However, the difference in PFS between surgical techniques was not statistically significant (log-rank *p* > 0.05), as shown in Figure 2.

In the multivisceral resection group with available follow-up, two patients (12.5%) did not receive any adjuvant therapy. Six patients (37.5%) received mitotane monotherapy, and seven (43.8%) received combined therapy with mitotane and etoposide, doxorubicin, and paclitaxel (EDP). One patient (6.3%) additionally received radiotherapy. None of the patients received a neoadjuvant therapy.

Among those who experienced disease recurrence in this group, two patients (12.5%) had a local recurrence, eight (50.0%) developed new distant metastases, and one had both. One patient (6.3%) had lymph node metastasis, and another (6.3%) had both new distant metastases and progression of previously known metastases. Among the nine patients with distant metastases, six (66.7%) had pulmonary metastases, two (22.2%) had liver metastases, and one (11.1%) had concurrent pulmonary, hepatic, and lymph node metastases.

In the isolated adrenalectomy group with available follow-up, four patients (22.2%) did not receive any adjuvant therapy. Eleven patients (61.1%) received mitotane monotherapy, one (5.6%) received mitotane and EDP, and one (5.6%) received mitotane and radiotherapy.

Among patients in this group who experienced disease recurrence (*n* = 7), one (14.3%) had a local recurrence, five (71.4%) developed new distant metastases, and one (14.3%) had both a new distant metastasis and lymph node metastasis. Among the six patients with distant metastases, four (66.7%) had pulmonary metastases, one (16.7%) had bone metastasis, and one (16.7%) had liver metastasis.

## 4. Discussion

In this retrospective cohort study, we analyzed outcomes in 21 patients who underwent multivisceral resection for suspected adrenocortical carcinoma (ACC). We compared those with histologically confirmed ACC (*n* = 18) to 19 patients who underwent isolated adrenalectomy for ACC. We found that patients in the multivisceral resection group had significantly larger and more advanced tumors, with a higher ENSAT stage and greater S-GRAS scores. Despite more aggressive disease features, R0 resection was achieved in over 70% of multivisceral cases. Importantly, lymph node metastases were identified exclusively in this group, highlighting the need of lymphadenectomy in advanced cases. Although multivisceral resections were associated with longer operative times and consistently required intensive postoperative care, complication rates and overall survival did not differ significantly between the groups. Histologic confirmation of true organ invasion was limited, underscoring the difficulty of preoperative assessment. These findings support the feasibility and potential oncologic benefit of multivisceral resection in selected patients with advanced ACC.

ACC presents unique diagnostic challenges, particularly in differentiating it preoperatively from benign or metastatic lesions. In our series, 3 of 21 patients who underwent multivisceral resection were ultimately diagnosed with non-ACC histologies (sarcoma, adrenal metastasis, ganglioneuroma), despite radiological suspicion. Yalon et al. [6] introduced a score based on identification of the following features: size, attenuation, thin and thick rim enhancement patterns, heterogeneity, calcification, necrosis, fat infiltration, and lymph node prominence for the differentiation of ACC from lipid-poor adrenal adenoma with 100% sensitivity and 80% specificity [6]. Furthermore, Mihai et al. [3] suggested that the boarder for suspicions of ACC should be elevated to 20 HU to improve specificity of this diagnostic tool [3]. While cross-sectional imaging cannot definitively confirm malignancy [1], and tumor biopsy is discouraged due to the risk of tumor dissemination and impaired resectability [3], advances in liquid biopsy, including circulating tumor cells, tumor DNA and specific microRNAs (miRNAs; e.g., miR-483-5p, miR-210), offer promising approaches for non-invasive diagnosis and monitoring of ACC [15,16,17,18]. These tools may improve preoperative diagnostic accuracy and help avoid unnecessary extensive surgeries for benign lesions, though prospective validation is needed.

Multivisceral resection was pursued in our cohort primarily due to suspected infiltration of adjacent organs or vessels, adhesions, and tumor thrombus. However, histopathological confirmation of organ invasion was limited—found only in the liver (2 cases) and diaphragm (1 case)—highlighting the challenge of preoperatively recognizing true invasion. Notably, no pathological evidence of kidney infiltration was found in any case, despite nephrectomy being performed in over half of the patients (52.4%). This observation supports the recommendation by Mihai et al. and that kidney preservation should be prioritized whenever oncologically safe, as the adrenal tumor rarely invades the kidney directly [3]. Furthermore, data by Propiglia et al. [19] reinforce this conclusion, showing that nephrectomy does not improve oncologic outcomes in patients with stage II ACC, and thus should be avoided in the absence of overt renal involvement [19]. In our cohort, R0 resection was still achieved in 72.2% of multivisceral resection cases, demonstrating that complete tumor removal is feasible in anatomically challenging cases. Compared to isolated adrenalectomy, patients undergoing multivisceral resection had significantly larger tumors, higher S-GRAS and ENSAT stage scores, and more frequent vascular invasion. These procedures required open or converted approaches, longer operative times, and extended ICU care. Despite these challenges, complication rates and overall survival did not significantly differ between multivisceral and isolated adrenalectomy groups, even though the former had significantly more advanced disease. Our findings are consistent with previous studies. Procopio et al. [20] demonstrated that stage III ACC patients who underwent extended en bloc R0 resections had overall and disease-free survival comparable to those with stage I/II disease, and without increased postoperative morbidity [20]. Furthermore, it had previously been shown, that stage IV patients with metastatic disease had a longer overall survival, if metastases were resected. Shariq et al. [21] reported that multivisceral resection was associated with longer median overall survival (median 33 months) compared to isolated adrenalectomy (median 22 months), with both outperforming systemic therapies alone [21]. Baur et al. [22] focused solely on ACC patients with liver metastasis and could show that while disease-free survival was short (median 9.1 months), the overall survival was significantly better in patients with resected vs. not resected metastases (median 76.1 vs. 10.1 months) [22]. These data, along with our findings, reinforce that multivisceral resection, when performed in high-volume centers, can offer meaningful oncologic benefit in selected patients, particularly when complete resection is achievable. 

Lymphnode metastases were revealed in one-third of patients in the multivisceral group. It is recommended to include locoregional lymph node dissection as part of surgery for suspected ACC, especially when preoperative cross-sectional imaging suggests nodal involvement [3,23]. The absence of nodal metastases in the isolated adrenalectomy group highlights the association between advanced disease and lymphatic spread. While the therapeutic benefit of lymphadenectomy remains debated, studies such as those by Reibetanz et al. [24] and Deschner et al. [25] emphasize its prognostic value. In our series, nodal dissection contributed to accurate staging and informed postoperative therapy decisions.

None of the patients in our cohort received neoadjuvant therapy prior to surgery. Neoadjuvant EDP-M therapy has shown potential to downstage tumors, facilitate R0 resection, and select biologically favorable tumors for surgery [1,26,27]. Given the limited progression-free survival observed in the multivisceral group (median 9.5 months), the potential role of preoperative systemic therapy in improving outcomes deserves exploration in future studies.

All multivisceral resections in this study were performed via open approaches, except for one case that required conversion from a minimally invasive approach. Although a laparoscopic approach for ENSAT stage I–III adrenocortical carcinoma (ACC) offers comparable oncologic outcomes to open surgery when an R0 resection is achieved [28], current guidelines discourage laparoscopy in cases of suspected local invasion [7]. While both laparoscopic [28] and retroperitoneoscopic [29] approaches are feasible for adrenalectomy in malignant tumors, robotic adrenalectomy may offer additional advantages in ACC surgery, particularly by reducing conversion rates [30]. Given the technical complexity of these procedures and the need for intensive postoperative care, current recommendations support performing ACC surgeries in specialized, high-volume centers [31,32,33]. In our series, high R0 resection rates and acceptable morbidity underscore the value of surgical expertise in optimizing outcomes.

This study has several limitations. First, the retrospective design raises the possibility of selection bias. Second, the study was conducted at a single tertiary referral center, which may affect the generalizability of the findings to other settings with varying surgical expertise and case volumes. Finally, the relatively small sample size, particularly within subgroup comparisons, limits statistical power and precludes multivariable analysis to adjust for potential confounding factors. Despite these limitations, the study provides valuable insights into the role and outcomes of multivisceral resection in the management of suspected ACC and highlights key areas for future multicentric prospective investigation.

## 5. Conclusions

Our study demonstrates that multivisceral resection for suspected or advanced adrenocortical carcinoma is feasible and can achieve high rates of complete (R0) resection, even in cases with aggressive disease features. Importantly, although baseline disease severity was consistently higher in patients requiring multivisceral resection, outcomes in terms of morbidity and overall survival were comparable to those of isolated adrenalectomy. This indicates that, in the hands of a skilled and highly experienced surgical team, multivisceral resection can offer prognostic results equivalent to single-adrenalectomy, thereby reinforcing surgery as the cornerstone of a multimodal treatment approach. Moreover, our findings underscore the importance of lymphadenectomy for accurate staging and highlight the ongoing need for improved preoperative diagnostic tools to optimize treatment of patients with other adrenal or retroperitoneal tumour entities. Future prospective, multicenter studies are needed to refine patient selection and clarify the role of neoadjuvant therapies in the management of this challenging malignancy.

## Figures and Tables

**Table 1:** Multivisceral resection for suspected adrenocortical carcinoma with curative intention—patient and tumour characteristics (*n* = 21).

Patient and Tumour Characteristics		
Gender ^1^	Female	14 (66.7%)
	Male	7 (33.3%)
Age (years) ^2^		42 (15)
BMI (kg/m^2^) ^3^ (n.a. = 1)		25.1 [18.2–78]
ASA ^1^	1	2 (9.5%)
	2	5 (23.8%)
	3	13 (61.9%)
	4	1 (4.8%)
Localization of the adrenal	Right	9 (42.9%)
tumour ^1^	Left	12 (57.1%)
Tumour size (mm) ^2^		142 (54)
Hormonal activity ^1^ (n.a. = 1)	Yes	15 (71.4%)
	No	6 (28.6%)
Tumour entity	ACC	18 (85.6%)
	Ganglioneuroma	1 (4.8%)
	Adrenal metastasis	1 (4.8%)
	Sarcoma	1 (4.8%)
Lymphnode metastases ^1^	Yes	6 (28.6%)
	No	15 (71.4%)
Vascular infiltration	Yes	11 (52.4%)
	No	10 (47.6%)
Macroscopic		5 (23.8%)
Microscopic		6 (28.6%)
Infiltration of adjacent solid	Yes	2 (10%)
organs on histology	No	18 (90%)
(per continuam)		
Liver		2 (9.5%)
Kidney		0 (0%)
Pancreas		0 (0%)
Diaphragm		1 (4.8%)
Spleen		0 (0%)

^1^ Count (percentage); ^2^ Mean (standard deviation); ^3^ Median [range]; n.a., not available; ACC, adrenocortical carcinoma.

**Table 2:** Multivisceral resection for suspected adrenocortical carcinoma with curative intention—intra- and postoperative outcomes (*n* = 21).

Intra- and Postoperative Course		
Indication to en bloc	Adhesions to adjacent organs	4 (19.1%)
resection ^1^	Suspected adjacent organ infiltration	4 (14.3%)
	Suspected infiltration of kidney vessels	7 (33.3%)
	Liver metastasis	4 (19.1%)
	Tumor thrombus in VCI	1 (4.8%)
	Achieving a better overview to enable R0 resection	1 (4.8%)
	Suspected adjacent organ infiltration and VCI	
	infiltration	1 (4.8%)
	Adhesions and VCI infiltration	1 (4.8%)
Extent of the en bloc	Kidney	6 * (28.6%)
resection ^1^	Liver	2 (9.5%)
	Spleen	1 (4.8%)
	Diaphragm	1 (4.8%)
	VCI Thrombectomy	1 (4.8%)
	Liver and gall bladder	3 (14.3%)
	Kidney, liver, gall bladder and VCI thrombectomy	1 (4.8%)
	Kidney and diaphragm	2 (9.5%)
	Pancreatic tail and spleen	2 (9.5%)
	Kidney and spleen	1 (4.8%)
	Kidney and VCI thrombectomy	1 (4.8%)
Technique ^1^	Open surgery	18 (85.7%)
	Hybrid approach (conversion)	3 (14.3%)
Duration of surgery (minutes) ^3^ (n.a. = 1)		236 [102–825]
Complete resection ^1^	Yes (R0)	16 (76.2%)
	No (R1)	1 (4.8%)
	Cannot be assessed (Rx)	4 (19%)
Blood transfusion ^1^	Yes	6 (28.6%)
	No	15 (71.4%)
ICU stay ^1^	Yes	20 (95.2%)
	No	1 (4.8%)
Length of postoperative ICU stay ^3^		1 [1–38]
Length of postoperative hospital stay ^3^		12 [6–78]
Complications (any) ^1^	Yes	9 (42.9%)
	No	12 (57.1%)
Type of complications ^1^	Minor	6 (28.6%)
	Major	3 (14.3%)

^1^ Count (percentage); ^3^ Median [range]; n.a., not available; VCI, vena cava inferior; ICU, Intensive Care Unit; * one partial adrenalectomy.

**Table 3:** Comparison of patient and tumour characteristics in patients undergoing multivisceral resection and isolated adrenalectomy for ACC with curative intention (*n* = 37).

	**Multivisceral Resection (*n* = 18)**	**Isolated Adrenalectomy (*n* = 19)**	***p*-Value**
Gender ^1^			
Female	13 (72.2%)	12 (63.2%)	0.728
Male	5 (27.8%)	7 (36.8%)	
Age (years) ^2,3^	43 (16)	64 [26–77]	0.003
BMI (kg/m^2^) ^3^	29 (14)	29 (6)	0.7831
	(n.a. = 1)	(n.a. = 2)	
ASA ^1^			
1	0 (0%)	2 (10.5%)	
2	5 (27.8%)	10 (52.6%)	0.0808
3	12 (66.7%)	7 (36.9%)	
4	1 (5.5%)	0 (0%)	
Tumour side ^1^			
Right	7 (38.9%)	13 (68.4%)	0.103
Left	11 (61.1%)	6 (31.6%)	
Tumour size (mm) ^3^	149 (53)	53 (36)	<0.001
Hormonal activity ^1^	14 (77.8%)	12 (63%)	0.274
	(n.a. = 1)		
S-GRAS score ^1^			
0–3	2 (11.1%)	10 (52.6%)	0.0128
4–8	16 (88.9%)	9 (37.4%)	
ENSAT stage ^1^			
I and II	2 (11.1%)	15 (78.9%)	<0.001
III	11 (61.1%)	4 (21.1%)	
IV	5 (27.8%)	0 (0%)	
Lymph node metastases ^1^	6 (33.3%)	0 (0%)	0.008
Vascular infiltration ^1^	9 (50%)	8 (42.1%)	0.746
Ki67-Index (in%) 2	13.5 [5–70]	11 [3–60]	0.902

^1^ Count (percentage); ^2^ Mean (standard deviation); ^3^ Median [range]; n.a., not available; S-GRAS, tumor stage, grade, resection status, age, symptoms; ENSAT, European Network for the Study of Adrenal Tumors.

**Table 4:** Comparison of intra- and postoperative outcomes in patients undergoing multivisceral resection and isolated adrenalectomy for ACC with curative intention (*n* = 37).

	**Multivisceral Resection (*n* = 18)**	**Isolated Adrenalectomy (*n* = 19)**	***p*-Value**
Technique ^1^			
Laparoscopic	0 (0%)	8 (42.1%)	
Open	17 (94.4%)	6 (31.6%)	<0.001
Hybrid	1 (5.6%)	5 (26.3%)	
(conversion)			
Duration of surgery (minutes) ^2^	243 [102–825]	139 [73–152]	
	(n.a. =1)	(n.a. = 1)	0.002
Resection status ^1^			
R0	13 (72.2%)	16 (84.2%)	
R1	1 (5.6%)	3 (15.8%)	0.081
Rx	4 (22.2%)	0 (0%)	
Blood transfusion ^1^	6 (33.3%)	2 (10.5%)	0.125
	12 (66.6%)	17 (89.5%)	
ICU stay ^1^	18 (100%)	8 (42.1%)	<0.001
Length of postoperative ICU stay (days) ^2^	1 [1–38]	1 [0.5–2]	0.0528
Length of postoperative hospital stay (days) ^2^	15 [6–78]	6 [2–40]	0.001
Complications (any) ^1^	9 (50%)	4 (21%)	0.091
Major complications ^1^	3 (16.7%)	0 (0%)	0.105
Minor complications ^1^	6 (33.3%)	4 (21%)	0.476

^1^ Count (percentage); ^2^ Mean (standard deviation); n.a., not available; ICU, intensive care unit.

## Data Availability

The datasets generated during and analyzed during the current study are not publicly available due to reasons of sensitivity.

## Abbreviations

The following abbreviations are used in this manuscript:

ACC	Adrenocortical carcinoma
ENSAT	European Network for the Study of Adrenal Tumors
ICU	Intensive Care Unit
CT	Computed tomography
HU	Hounsfield Units
ASA	American Society of Anesthesiologists
BMI	Body Mass Index
VCI	Vena cava inferior
S-GRAS	tumor stage, grade, resection status, age, symptoms
EDP	etoposide, doxorubicin, paclitaxel
PFS	Progression-free survival
OS	Overall survival
DNA	Deoxyribonucleic acid
miRNA	microRNA
EDP-M	Etoposide, doxorubicin, paclitaxel—mitotane

## Appendix A

**Table A1:** Shapiro–Wilk test results assessing data distribution in patients with suspected adrenocortical carcinoma.

	Multivisceral Resection for Suspected ACC (*n* = 21)	
Variable	*p*-Value	Distribution
Age (years)	0.806	Normal
BMI (kg/m^2^) (n.a. = 1)	<0.001	Non-normal
Tumour size (mm)	0.453	Normal
Duration of surgery (minutes)	<0.001	Non-normal
Length of postoperative ICU stay	<0.001	Non-normal
Length of postoperative hospital stay	<0.001	Non-normal

ACC, adrenocortical carcinoma; BMI, body mass index; ICU, intensive care unit; n.a., not available.

**Table A2:** Shapiro–Wilk test results assessing data distribution in patients with confirmed adrenocortical carcinoma.

	Multivisceral Resection (*n* = 18)		Isolated Adrenalectomy (*n* = 19)	
Variable	*p*-Value	Distribution	*p*-Value	Distribution
Age (years)	0.919	Normal	0.02732	Non-normal
BMI (kg/m^2^) (n.a. = 1)	0.629	Normal	0.590	Normal
Tumour size (mm)	0.579	Normal	0.314	Normal
Ki67	<0.001	Non-normal	0.003	Non-normal
Duration of surgery (min)	0.002	Non-normal	0.017	Non-normal
Length of postoperative ICU stay	<0.001	Non-normal	0.002	Non-normal
Length of postoperative hospital stay	<0.001	Non-normal	<0.001	Non-normal

BMI, body mass index; ICU, intensive care unit; n.a., not available.

**Table A3:** Overview of hormonal oversecretion by adrenal tumors.

Hormonal Secretion	Multivisceral Resection (*n* = 18)	Isolated Adrenalectomy (*n* = 19; n.a. = 1)
None	4 (22.2%)	6 (33.3%)
Aldosterone	0 (0%)	2 (11.1%)
Cortisole	3 (16.7%)	3 (16.7%)
Androgen		
17-OH-Progesterone	2 (11.1%)	1 (5.5%)
DHEAS	2 (11.1%)	0 (0%)
Androstendione	1 (5.5%)	0 (0%)
17-OH-Progesterone, DHEAS and	1 (5.5%)	0 (0%)
Oestradiole		
Cortisole and Androgen		
Cortisol and 17-OH-Progesterone	0 (0%)	1 (5.5%)
Cortisole and DHEAS	1 (5.5%)	0 (0%)
Cortisole and Androstendione	1 (5.5%)	0 (0%)
Cortisole, 17-OH-Progesterone and DHEAS	0 (0%)	2 (11.1%)
Cortisole, 17-OH-Progesterone and Androstendione	3 (16.7%)	0 (0%)
Cortisole, 17-OH-Progesterone, DHEAS and Androstendione	0 (0%)	3 (16.7%)

DHEAS, dehydroepiandrosterone sulfate; n.a., not available.
